# Social Isolation and All-Cause and Heart Disease Mortality Among Working-Age Adults in the United States: The 1998–2014 NHIS–NDI Record Linkage Study

**DOI:** 10.1089/heq.2021.0003

**Published:** 2021-10-25

**Authors:** Hyunjung Lee, Gopal K. Singh

**Affiliations:** ^1^ Oak Ridge Institute for Science and Education (ORISE), Oak Ridge, Tennessee, USA.; ^2^ US Department of Health and Human Services, Health Resources and Services Administration, Office of Health Equity, Rockville, Maryland, USA.

**Keywords:** social isolation, living alone, social determinants, all-cause mortality, heart disease mortality, NHIS–NDI

## Abstract

**Purpose:** Living alone, an indicator of social isolation, has been increasing in the United States; 28% of households in 2019 were one-person households, compared with 13% in 1960. The working-age population is particularly vulnerable to adverse social conditions such as low social support. Although previous research has shown that social isolation and loneliness lead to poorer health and decreased longevity, few studies have focused on the working-age population and heart disease mortality in the United States using longitudinal data.

**Methods:** This study examines social isolation as a risk factor for all-cause and heart disease mortality among U.S. adults aged 18–64 years using the pooled 1998–2014 data from the National Health Interview Survey (NHIS) linked to National Death Index (NDI) (*n*=388,973). Cox proportional hazards regression was used to model survival time as a function of social isolation, measured by “living alone,” and sociodemographic, behavioral, and health characteristics.

**Results:** In Cox regression models with 17 years of mortality follow-up, the age-adjusted all-cause mortality risk was 45% higher (hazard ratio [HR]=1.45; 95% confidence interval [CI]=1.40–1.50) and the heart disease mortality risk was 83% higher (HR=1.83; 95% CI=1.67–2.00) among adults aged 18–64 years living alone at the baseline, compared with adults living with others. In the full model, the relative risk associated with social isolation was 16% higher (HR=1.16; 95% CI=1.11–1.20) for all-cause mortality and 33% higher (HR=1.33; 95% CI=1.21–1.47) for heart disease mortality after controlling for sociodemographic, behavioral-risk, and health status characteristics.

**Conclusion:** In this national study, adults experiencing social isolation had statistically significantly higher relative risks of all-cause and heart disease mortality in the United States than adults living with others.

## Introduction

Living alone, an indicator of social isolation, has been increasing in the United States; 28% of households in 2019 were one-person households, compared with 13% in 1960.^1^ The working-age population, compared with the general population, is more vulnerable to adverse social conditions such as low social support.^2,3^ Social isolation, loneliness, or living alone could influence mortality risk through behavioral, psychological, and physiological mechanisms.^4–8^ During working age, loneliness can negatively affect mental health, sleep quality, eating behavior, immunity, and proinflammatory response to stress, resulting in an increase in mortality.^5,8^

Living alone has been studied as a determinant of all-cause mortality and cardiovascular disease incidence and mortality.^3,8–14^ Most studies have examined the association for older adults with a mean age of 66 years,^3,11,12^ and 7–8 years of follow-up.^3,10,12^ The mortality risk of living alone has been shown to vary by age and gender, with females and older adults having lower relative mortality risks.^3,11–13^

However, a few studies have reported mixed findings such as no statistically significant association between living alone and morbidity or mortality.^15,16^ Several studies, using a composite measure of social isolation, have found that social isolation and loneliness lead to poorer health and decreased longevity,^3,17–22^ but few studies have examined the relationship between social isolation and heart disease mortality among the working-age population in the United States using longitudinal data,^17,20^ and differentiated by gender or race/ethnicity.^17,20,21^

In short, although previous research has examined the association between social isolation and mortality, the association has not been explored in detail, and there is limited research in the United States on living alone among working-age adults with long follow-up and stratified by gender and race/ethnicity. To address this gap in research, we examined the association between living alone and relative risks of all-cause and heart disease mortality in the United States using a nationally representative data set with 17 years of mortality follow-up.

## Methods

### Data

The data for this study are derived from the National Health Interview Survey (NHIS) linked to the National Death Index (NDI).^23^ As a nationally representative annual cross-sectional household interview survey, NHIS provides demographic, socioeconomic, and health characteristics of the civilian noninstitutionalized population in the United States. The National Center for Health Statistics (NCHS) developed public-use versions of NHIS linked with death certificate records from the NDI. For this study, we used the 1998–2014 public-use linked mortality file containing 17 years of mortality follow-up data from the date of survey participation through December 31, 2014.^24^ The study was exempt from Institutional Review Board approval as it utilized a de-identified public use dataset.

### Sample

The study sample was restricted to adults aged 18–64 from the years of 1998 to 2014 NHIS sample adult files. The records ineligible for mortality follow-up were eliminated from the analysis. The final pooled eligible sample size was 388,973. For missing values for poverty status (13.55%), psychological distress (1.17%), body mass index (BMI) (3.12%), and alcohol consumption (1.14%), we created missing covariate categories to prevent omission of many observations from the analysis.

### Measurement

Our outcomes of interest were all-cause mortality and heart disease mortality (International Classification of Diseases-10 codes: I00–I09, I11, I13, I20–I51). Follow-up time for individuals who died during the study period was estimated by the number of months from the month/year of interview to the month/year of death. Since the NHIS–NDI database provides only the quarter of death, we assumed that deaths occurred in the middle of the quarter, February, May, August, or November.^25^

### Living alone

For the measure of living arrangement, we used the family structure, a derived variable from familial relationship status and parental marital status if children are present, as hereunder. Respondents reporting living alone for the family structure variable were categorized as *living alone*. All other responses were categorized as *living with others*.

- *Living alone*
- *Living with roommate*
- *Married couple*
- *Unmarried couple*
- *All other adult only families*
- *Mother and biological or nonbiological children only*
- *Father and biological or nonbiological children only*
- *All other single-adult and children families*
- *Married or unmarried parents with biological/adoptive children only*
- *Parent (biological or adoptive), step parent, and children only*
- *Parent (biological or adoptive), cohabiting partner, and children only*
- *At least one (biological or adoptive) parent, more than one child, and other related adults*
- *Other related and/or unrelated adults, more than one child (no biological or adoptive parent)*

Living alone has been widely used as an objective measure of social isolation in empirical research.^9–12,15,26^ Although social isolation scale, social network index, and loneliness are also widely used to examine the association between social isolation and mortality, there are few differences among the weighted mean effect sizes by the three measures, ranging from 1.26 to 1.83.^3^

### Covariates

Based on the previous literature, we selected the following covariates for model estimation: age, gender, race/ethnicity, nativity/immigrant status, education, poverty status, housing tenure, region of residence, self-reported health status, activity limitation, psychological distress, BMI, smoking status, alcohol consumption, and survey years.^25,27–31^ These covariates were measured as given in Table 1. Age was categorized into nine 5-year age groups: 18–24, 25–29, 30–34, 35–39, 40–44, 45–49, 50–54, 55–59, and 60–64. Race/ethnicity consisted of non-Hispanic Whites, non-Hispanic Blacks, Hispanics, American Indians/Alaska Natives (AIANs), Asian and Pacific Islanders (APIs), and non-Hispanic other races.

**Table 1.** Descriptive Statistics for Individual Characteristics Among U.S. Adults Aged 18–64 Years, 1998–2014

	Total	Living with others	Living alone	*p*
Sample size (*n*, weighted %)	388,973	296,999 (86.24)	91,974 (13.76)	
All-cause deaths	19,792	13,104 (79.56)	6,688 (20.44)	<0.001
Heart disease deaths	2,776	1,627 (74.86)	1,149 (25.14)	<0.001
Age (%)				
18–24	15.56	89.22	10.78	<0.001
25–29	10.73	85.96	14.04	
30–34	11.00	88.18	11.82	
35–39	11.23	89.15	10.85	
40–44	11.91	88.55	11.45	
45–49	11.65	86.06	13.94	
50–54	10.94	83.67	16.33	
55–59	9.13	81.44	18.56	
60–64	7.85	79.81	20.19	
Gender (%)				
Male	49.10	84.33	15.67	<0.001
Female	50.90	88.09	11.91	
Race/ethnicity (%)				
Non-Hispanic White	68.47	85.84	14.16	<0.001
Non-Hispanic Black	12.12	80.91	19.09	
Hispanic	14.04	91.96	8.04	
American Indian/Alaska Native	0.69	85.86	14.14	
Asian/Pacific Islander	4.35	89.18	10.82	
Non-Hispanic other	0.33	85.29	14.71	
Nativity/immigrant status (%)				
Foreign born	16.32	90.68	9.32	<0.001
U.S. born	83.68	85.38	14.62	
Education (%)				
Less than high school	11.77	89.77	10.23	<0.001
High school	29.82	88.20	11.80	
Some college	31.27	85.51	14.49	
College	27.14	83.41	16.59	
Poverty status (%)				
<100	10.89	78.26	21.74	<0.001
≥100 and <200	14.18	85.70	14.30	
≥200 and <400	25.74	85.78	14.22	
≥400	34.98	88.17	11.83	
Unknown/missing	14.22	89.01	10.99	
Housing tenure (home ownership) (%)				
Renter	33.94	76.78	23.22	<0.001
Owner	66.06	91.11	8.89	
Region (%)				
Northeast	17.86	86.04	13.96	<0.001
Midwest	24.26	85.64	14.36	
South	36.58	86.13	13.87	
West	21.30	87.30	12.70	
Self-reported health status (%)				
Excellent/very good/good	89.86	86.80	13.20	<0.001
Fair/poor	10.14	81.33	18.67	
Activity limitation (%)				
No	88.55	87.46	12.54	<0.001
Yes	11.45	76.84	23.16	
K6 score (psychological distress (%)				
0	47.15	87.72	12.28	<0.001
1–2	20.69	86.38	13.62	
3–5	15.69	85.46	14.54	
6–12	12.05	83.28	16.72	
13–24	3.24	79.56	20.44	
Missing	1.18	83.75	16.25	
BMI (%)				
<25	38.14	85.80	14.20	<0.001
≥25 and <30	33.42	86.40	13.60	
≥30 and <40	21.65	86.89	13.11	
≥40	3.76	84.78	15.22	
Unknown/missing	3.03	87.30	12.70	
Smoking status (%)				
Never smoker	58.42	87.57	12.43	<0.001
Former smoker	18.51	85.99	14.01	
Current smoker	23.08	83.10	16.90	
Alcohol consumption (%)				
Lifetime abstainer	20.25	90.16	9.84	<0.001
Former drinker	12.25	85.77	14.23	
Current drinker	66.38	85.21	14.79	
Unknown/missing	1.11	81.84	18.16	

Source: Data derived from the 1998–2014 NHIS–NDI record linkage study.

BMI, body mass index; K6, Kessler 6; NHIS–NDI, National Health Interview Survey-National Death Index.

Nativity/immigrant status was categorized as U.S. born (born in one of the 50 states or the District of Columbia) or foreign born. Educational attainment was defined by four categories as less than high school diploma, high school diploma, some college, and college degree or more. Marital status was categorized as currently married, widowed, divorced/separated, and never married. Poverty status was defined by five categories based on the ratio of family income to poverty threshold (<100%, ≥100% and <200%, ≥200% and <400%, ≥400%, and missing). Housing tenure was dichotomized, with 1 being renters and 0 equaling homeowners. Region of residence was defined by four categories: northeast, midwest, south, and west.

Self-reported health status was dichotomized, with 1 being fair or poor health and 0 being excellent, very good, or good health. Activity limitation was defined as 1 for the person having an activity limitation listed in the NHIS survey questionnaire including instrumental activities of daily living, activities of daily living, working at a job, walking, or remembering, and 0 otherwise. Psychological distress was measured by the Kessler 6 (K6) nonspecific distress scale^32^ of six symptoms, ranging in value from 0 to 24. We created a five-level categorical variable with scores of 0, 1–2, 3–5, 6–12, and 13–24 from the K6 scale; a score of 13 or higher was defined as serious psychological distress (SPD).^28^

BMI was defined by five categories: <25, 25–29, 30–39, ≥40, and missing. Smoking status was defined by three categories as never, former, and current smokers. Alcohol consumption was defined by five categories: lifetime abstainer, former drinker, current drinker, and unknown.

### Analytic approach

Cox proportional hazards models were used to derive relative risks of all-cause and heart disease mortality, controlling for individual characteristics and year-fixed effects. The model assumes that hazard rates are a log-linear function of parameters representing the effects of covariates.^33,34^ Individuals surviving beyond the follow-up period and those dying from causes other than heart disease were treated as right-censored observations. The Cox models were estimated separately for females and males and for different racial/ethnic groups. Group differences were manually calculated using coefficients and standard errors.

We checked the hazards proportionality assumption^34^ by inspecting the plots of ln(-ln{S(*t*)]) [log(-log) survival function] against survival time *t* for the various covariate categories including those for living alone, gender, race/ethnicity, education, poverty level, activity limitation, psychological distress, and self-reported health status.^35^ These plots were found to be approximately parallel and hence the proportionality assumption was taken to be satisfied by the data.

Complex survey design procedures were used to account for clustering, multiple stages of selection, and disproportionate sampling. To correct the bias from the ineligible adults for linkage to the NDI due to insufficient identifying data, we used eligibility-adjusted weights developed by NCHS, instead of the standard sample weight.^36^ The sample weights were adjusted by dividing by the number of pooling years. All analyses were conducted by Stata 16^37^ and the Cox model was fitted using *stcox* procedure.

We estimated a survival function derived from fully adjusted Cox proportional hazard regression models of all-cause mortality and a cumulative incidence function derived from fully adjusted competing-risks regression of heart disease mortality, based on the method of Fine and Gray,^38^ by living arrangement and gender.

## Results

### Descriptive statistics

Table 1 provides descriptive statistics for individual characteristics by living arrangements. Approximately 13.76% of the respondents lived alone. The proportion living alone was highest among adults aged 60–64 years, males, non-Hispanic Blacks, the U.S. born, those with college degree, those below the poverty level, renters, residents of the midwest, those with fair or poor self-reported health status, those with activity limitation, those with SPD, those with severe obesity (BMI ≥40), current smokers, and current drinkers. The total number of all-cause deaths and heart disease deaths during the 17-year follow-up was 19,792 and 2,776, respectively.

### Cox proportional hazards models

In Cox regression model 1, controlling for age and survey year, the all-cause mortality risk was 45% (hazard ratio [HR]=1.45; 95% confidence interval [CI]=1.40–1.50) higher in adults living alone (*p*<0.001) at the baseline, compared with adult living with others (Table 2). In model 2, after controlling for age, survey year, and socioeconomic and demographic characteristics, the all-cause mortality risk was 24% higher (HR=1.24; 95% CI=1.20–1.29) in adults living alone (*p*<0.001), compared with adult living with others.

**Table 2.** Age–Year-Adjusted and Covariate-Adjusted Hazard Ratios of All-Cause Mortality by Social Isolation Among U.S. Adults Aged 18–64 Years, 1998–2014 (*n*=388,973)

Covariates	Age–year-adjusted model^a^	Sociodemographically adjusted model^b^	Fully adjusted model^c^
All-cause mortality			
Living with others	1.00	1.00	1.00
Living alone	1.45 (1.40–1.50)^***^	1.24 (1.20–1.29)^***^	1.16 (1.11–1.20)^***^
Heart disease mortality			
Living with others	1.00	1.00	1.00
Living alone	1.83 (1.67–2.00)^***^	1.41 (1.28–1.55)^***^	1.33 (1.21–1.47)^***^
Gender			
Male		1.00	1.00
Female		0.62 (0.60–0.64)^***^	0.63 (0.61–0.65)^***^
Race/ethnicity			
Non-Hispanic White		1.00	1.00
Non-Hispanic Black		1.15 (1.10–1.21)^***^	1.18 (1.13–1.24)^***^
Hispanic		1.13 (1.06–1.20)^***^	1.29 (1.21–1.37)^***^
American Indian/Alaska Native		1.33 (1.12–1.58)^**^	1.14 (0.96–1.36)
Asian/Pacific Islander		1.56 (1.39–1.74)^***^	1.52 (1.36–1.70)^***^
Non-Hispanic other		1.03 (0.67–1.58)	0.98 (0.64–1.50)
Nativity/immigrant status			
U.S. born		1.00	1.00
Foreign born		0.74 (0.69–0.78)^***^	0.93 (0.88–0.99)^*^
Education			
Less than high school		2.35 (2.20–2.51)^***^	1.49 (1.4–1.60)^***^
High school		1.77 (1.67–1.87)^***^	1.37 (1.30–1.45)^***^
Some college		1.49 (1.41–1.57)^***^	1.23 (1.16–1.30)^***^
College		1.00	1.00
Poverty status (%)			
<100		2.11 (1.98–2.25)^***^	1.26 (1.18–1.34)^***^
≥100 and <200		1.84 (1.74–1.95)^***^	1.29 (1.21–1.36)^***^
≥200 and <400		1.34 (1.28–1.42)^***^	1.15 (1.09–1.21)^***^
≥400		1.00	1.00
Unknown/missing		1.42 (1.34–1.49)^***^	1.20 (1.13–1.27)^***^
Housing tenure (home ownership)			
Renter		1.25 (1.20–1.31)^***^	1.09 (1.05–1.14)^***^
Owner		1.00	1.00
Region			
Northeast		0.93 (0.87–0.99)^*^	0.94 (0.89–1.00)^*^
Midwest		0.97 (0.92–1.03)	0.96 (0.90–1.01)
South		1.08 (1.02–1.14)^**^	1.04 (0.99–1.10)
West		1.00	1.00
Self-reported health status			
Fair/poor			1.74 (1.66–1.83)^***^
Excellent/very good/good			1.00
Activity limitation			
No			1.00
Yes			1.92 (1.84–2.01)^***^
K6 score (psychological distress)			
0			1.00
1–2			0.93 (0.88–0.98)^**^
3–5			1.00 (0.95–1.05)
6–12			1.02 (0.97–1.07)
13–24			0.98 (0.91–1.06)
missing			1.10 (0.97–1.25)
BMI			
<25			1.00
≥25 and <30			0.89 (0.85–0.93)^***^
≥30 and <40			0.98 (0.94–1.03)
≥40			1.29 (1.19–1.39)^***^
Unknown/missing			0.93 (0.83–1.05)
Smoking status			
Never smoker			1.00
Former smoker			1.25 (1.19–1.31)^***^
Current smoker			1.98 (1.90–2.06)^***^
Alcohol consumption			
Lifetime abstainer			1.00
Former drinker			1.02 (0.97–1.08)
Current drinker			0.83 (0.80–0.87)^***^
Unknown/missing			0.94 (0.80–1.11)

Source: Data derived from the 1998–2014 NHIS–NDI record linkage study.

^a^ Age–year-adjusted model is the Cox proportional hazards model adjusted for age and survey year.

^b^ Sociodemographically adjusted model is adjusted for age, survey year, gender, race/ethnicity, nativity/immigrant status, education, poverty status, housing tenure, and region.

^c^ Fully adjusted model is adjusted for age, survey year, gender, race/ethnicity, nativity/immigrant status, education, poverty status, housing tenure, region, self-assessed health status, activity limitation, psychological distress, BMI, smoking status, and alcohol consumption.

^***^ *p*<0.001, ^**^p<0.01, ^*^p<0.05.

In model 3, after controlling for all covariates including age, survey year, gender, race/ethnicity, nativity/immigrant status, education, poverty status, housing tenure, region, self-assessed health status, activity limitation, psychological distress, BMI, smoking status, and alcohol consumption, the all-cause mortality risk was 16% higher (HR=1.16; 95% CI=1.11–1.20) in adults living alone (*p*<0.001), compared with adults living with others. Heart disease mortality risk of living alone was greater than that of all-cause mortality.

Heart disease mortality risk of living alone was 83% higher (HR=1.83; 95% CI=1.67–2.00) in the age–year-adjusted model, 41% higher (HR=1.41; 95% CI=1.28–1.55) in the SES-adjusted model, and 33% higher (HR=1.33; 95% CI=1.21–1.47) in the fully adjusted model, compared with adults living with others.

### Differential mortality effects of living alone by gender and race/ethnicity

Table 3 provides differential effects of living alone on all-cause and heart disease morality by gender and race/ethnicity.

**Table 3.** Age–Year-Adjusted and Covariate-Adjusted Hazard Ratios and 95% Confidence Intervals for All-Cause and Heart Disease Mortality by Social Isolation Among U.S. Adults Aged 18–64 Years by Gender and Race/Ethnicity, 1998–2014

	All-cause mortality				Heart disease mortality	
	Age–year-adjusted model^a^	Sociodemographically adjusted model^b^	Fully adjusted model^c^	Age–year-adjusted model^a^	Sociodemographically adjusted model^b^	Fully adjusted model^c^
Male (*n*=175,979)						
Living with others	1.00	1.00	1.00	1.00	1.00	1.00
Living alone	1.48 (1.41–1.55)^***^	1.29 (1.23–1.35)^***^	1.20 (1.15–1.27)^***^	1.78 (1.60–1.99)^***^	1.41 (1.25–1.59)^***^	1.34 (1.19–1.51)^***^
Female (*n*=212,994)						
Living with others	1.00	1.00	1.00	1.00	1.00	1.00
Living alone	1.39 (1.32–1.47)^***^†^d^	1.19 (1.12–1.26)^***^†	1.09 (1.03–1.16)^**^†	1.95 (1.66–2.29)^***^	1.43 (1.19–1.72)^***^	1.33 (1.11–1.60)^**^
Non-Hispanic White (*n*=235,095)						
Living with others	1.00	1.00	1.00	1.00	1.00	1.00
Living alone	1.57 (1.50–1.64)^***^	1.29 (1.23–1.36)^***^	1.22 (1.17–1.28)^***^	1.83 (1.64–2.04)^***^	1.40 (1.24–1.58)^***^	1.34 (1.19–1.51)^***^
Non-Hispanic Black (*n*=58,417)						
Living with others	1.00	1.00	1.00	1.00	1.00	1.00
Living alone	1.20 (1.11–1.29)^***^†	1.05 (0.97–1.14)	1.01 (0.93–1.10)	1.50 (1.23–1.84)^***^†	1.27 (1.03–1.56)^**^	1.23 (1.00–1.51)
Hispanic (*n*=73,805)						
Living with others	1.00	1.00	1.00	1.00	1.00	1.00
Living alone	1.05 (0.94–1.16)	1.08 (0.97–1.20)	0.99 (0.89–1.10)	1.59 (1.19–2.13)^**^	1.51 (1.13–2.02)^**^	1.43 (1.05–1.96)^*^
Non-Hispanic other (*n*=21,656)						
Living with others	1.00	1.00	1.00	1.00	1.00	1.00
Living alone	1.29 (1.07–1.56)^**^†	1.19 (0.99–1.43)	1.12 (0.93–1.35)	2.57 (1.64–4.02)^***^	1.88 (1.17–3.04)^*^	1.76 (1.08–2.85)^*^

Source: Data derived from the 1998–2014 NHIS–NDI record linkage study.

^a^ Age–year-adjusted model is the Cox proportional hazards model adjusted for age and survey year.

^b^ Sociodemographically adjusted model is adjusted for age, survey year, gender (excluded in gender -specific model), race/ethnicity (excluded in racial/ethnic-specific model), nativity/immigrant status, education, poverty status, housing tenure, and region.

^c^ Fully adjusted model is adjusted for age, survey year, gender (excluded in gender-specific model), race/ethnicity (excluded in racial/ethnic-specific model), nativity/immigrant status, education, poverty status, housing tenure, region, self-assessed health status, activity limitation, psychological distress, BMI, smoking status, and alcohol consumption.

^d^ Denotes statistical significance of the difference in hazard coefficients between males and females, non-Hispanic Whites and non-Hispanic Blacks.

^***^ *p*<0.001, ^**^p<0.01, ^*^p<0.05, ^†^*p*<0.05.

For males, the all-cause mortality risk was 48% higher (HR=1.48; 95% CI=1.41–1.55) in the age-adjusted model, 29% higher (HR=1.29; 95% CI=1.23–1.35) in the sociodemographically adjusted model, and 20% higher (HR=1.20; 95% CI=1.15–1.27) in the fully adjusted model among adults living alone (*p*<0.001) at the baseline, compared with adults living with others. Heart disease mortality risk was 78% higher (HR=1.78; 95% CI=1.60–1.99) in the age-adjusted model, 41% higher (HR=1.41; 95% CI=1.25–1.59) in the sociodemographically adjusted model, and 34% higher (HR=1.34; 95% CI=1.19–1.51) in the fully adjusted model among male adults living alone (*p*<0.001), compared with male adults living with others.

For females, the all-cause mortality risk was 39% higher (HR=1.39; 95% CI=1.32–1.47) in the age-adjusted model, 19% higher (HR=1.19; 95% CI=1.12–1.26) in the sociodemographically adjusted model, and 9% higher (HR=1.09; 95% CI=1.03–1.16) in the fully adjusted model among adults living alone (*p*<0.01), compared with adults living with others. Heart disease mortality risk was 95% higher (HR=1.95; 95% CI=1.66–2.29) in the age-adjusted model, 43% higher (HR=1.43; 95% CI=1.19–1.72) in the sociodemographically adjusted model, and 33% higher (HR=1.33; 95% CI=1.11–1.60) in the fully adjusted model among female adults living alone (*p*<0.01), compared with female adults living with others.

For non-Hispanic Whites, the all-cause mortality risk was 57% higher (HR=1.57; 95% CI=1.50–1.64) in the age-adjusted model, 29% higher (HR=1.29; 95% CI=1.23–1.36) in the sociodemographically adjusted model, and 22% higher (HR=1.22; 95% CI=1.17–1.28) in the fully adjusted model among adults living alone (*p*<0.001), compared with adults living with others. However, for racial/ethnic minorities, living alone was not statistically significantly associated with all-cause mortality, except for non-Hispanic Blacks and the other racial/ethnic groups (consisting mainly of APIs and AIANs) in the age-adjusted model.

Heart disease mortality risk for non-Hispanic Whites was 83% higher (HR=1.83; 95% CI=1.64–2.04) in the age-adjusted model, 40% higher (HR=1.40; 95% CI=1.24–1.58) in the sociodemographically adjusted model, and 34% higher (HR=1.34; 95% CI=1.19–1.51) in the fully adjusted model among adults living alone (*p*<0.001) at the baseline, compared with adults living with others. For non-Hispanic Blacks, heart disease mortality risk was 50% higher (HR=1.50; 95% CI=1.23–1.84) in the age-adjusted model and 27% higher (HR=1.27; 95% CI=1.03–1.56) in the sociodemographically adjusted model among adults living alone (*p*<0.01), compared with adults living with others, but was not statistically significant in the fully adjusted model.

For Hispanics, heart disease mortality risk was 59% higher (HR=1.59; 95% CI=1.19–2.13) in the age-adjusted model, 51% higher (HR=1.51; 95% CI=1.13–2.02) in the sociodemographically adjusted model, and 43% higher (HR=1.43; 95% CI=1.05–1.96) in the fully adjusted model among adults living alone (*p*<0.05), compared with adults living with others. For non-Hispanic other races, heart disease mortality risk was 157% higher (HR=2.57; 95% CI=1.64–4.02) in the age-adjusted model, 88% higher (HR=1.88; 95% CI=1.17–3.04) in the sociodemographically adjusted model, and 76% higher (HR=1.76; 95% CI=1.08–2.85) in the fully adjusted model among adults living alone (*p*<0.05), compared with adults living with others.

### Adjusted survival probabilities among males and females living alone

**FIG. 1.**
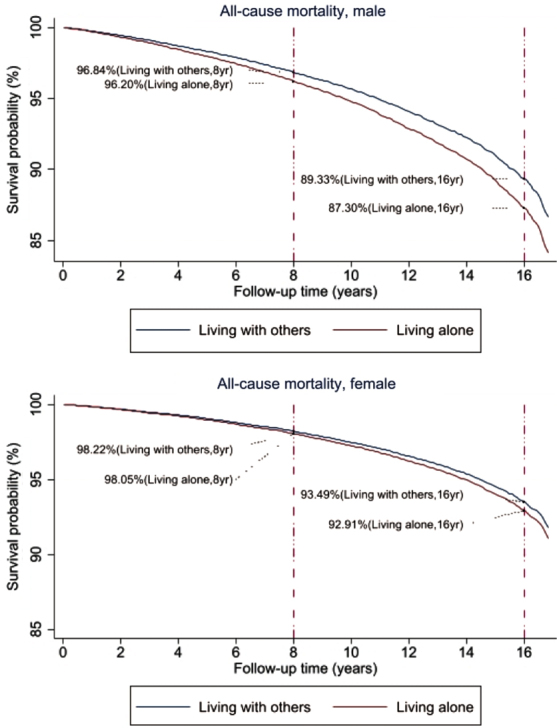
Adjusted survivorship of U.S. adults aged 18–64 years by living arrangement/social isolation and gender, 1998–2014 (derived from fully adjusted Cox models of all-cause mortality).

Figure 1 presents estimated survivor probabilities by social isolation using the fully adjusted Cox models for males and females computed at the mean values of other covariates. The estimated survival functions for all causes combined were steeper for males than for females. At the end of the 16-year follow-up, 87.3% of males living alone were expected to survive, compared with 89.3% of males living with others. The survival rates for females living alone were 92.9%, compared with 93.5% at the end of the 16-year follow-up.

**FIG. 2.**
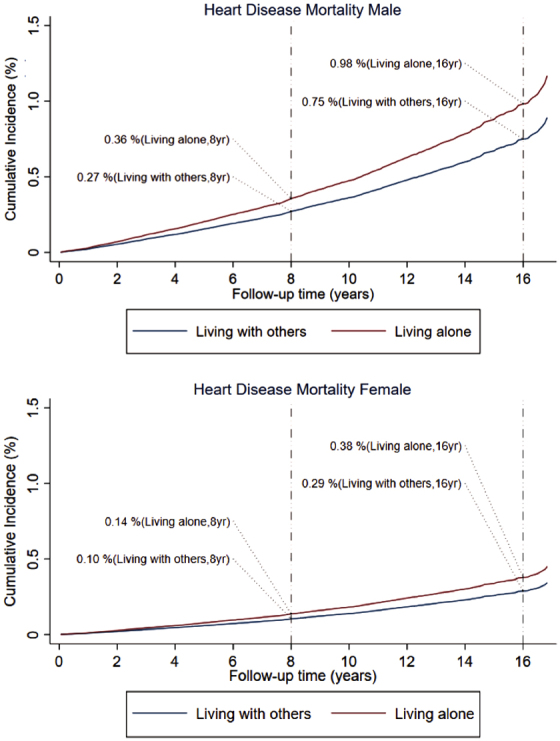
Cumulative mortality incidence function for U.S. adults aged 18–64 years by living arrangement/social isolation and gender, 1998–2014 (derived from fully adjusted, competing-risks regression of heart disease mortality, based on the method of Fine and Gray^38^).

Figure 2 presents the estimated cumulative incidence function by social isolation using the fully adjusted competing-risk regression of heart disease mortality for males and females. At the end of the 16-year follow-up, 0.98% of males living alone were expected to die from heart disease, compared with 0.75% of males living with others. The corresponding heart disease death rates for females living alone and living with others were 0.38% and 0.29%, respectively, at the end of the 16-year follow-up. Males living alone had significantly higher cumulative incidence rates of heart disease mortality than their female counterparts.

### Sensitivity analysis for testing temporal robustness of living alone

We also performed a sensitivity analysis to examine the temporal robustness of living alone effects by re-estimating Cox models using 2-, 5-, and 10-year follow-up times. As given in Table 4, HRs associated with living alone from various Cox models with different follow-up times remain stable, and hence HRs based on the 17-year mortality follow-up are not likely to be biased for all causes combined and for heart disease.

**Table 4.** Age–Year-Adjusted and Covariate-Adjusted Hazard Ratios and 95% Confidence Intervals for All-Cause and Heart Disease Mortality by Social Isolation by Different Follow-Up Times Among U.S. Adults Aged 18–64 Years, 1998–2014 (*n*=388,973)

Living arrangement/social isolation	Age–year-adjusted model^a^	Sociodemographically adjusted model^b^	Fully adjusted model^c^
All-cause mortality			
2-year mortality follow-up			
Living with others	1.00 (ref.)	1.00 (ref.)	1.00 (ref.)
Living alone	1.46 (1.34–1.60)^***^	1.22 (1.11–1.34)^***^	1.10 (1–10.21)
5-year mortality follow-up			
Living with others	1.00 (ref.)	1.00 (ref.)	1.00 (ref.)
Living alone	1.52 (1.43–1.61)^***^	1.26 (1.19–1.34)^***^	1.15 (1.09–1.23)^***^
10-year mortality follow-up			
Living with others	1.00 (ref.)	1.00 (ref.)	1.00 (ref.)
Living alone	1.49 (1.43–1.56)^***^	1.26 (1.21–1.32)^***^	1.17 (1.12–1.23)^***^
Heart disease mortality			
2-year mortality follow-up			
Living with others	1.00 (ref.)	1.00 (ref.)	1.00 (ref.)
Living alone	2.03 (1.64–2.53)^***^	1.47 (1.16–1.86)^**^	1.35 (1.06–1.73)^*^
5-year mortality follow-up			
Living with others	1.00 (ref.)	1.00 (ref.)	1.00 (ref.)
Living alone	1.87 (1.62–2.17)^***^	1.38 (1.18–1.62)^***^	1.29 (1.10–1.52)^**^
10-year mortality follow-up			
Living with others	1.00 (ref.)	1.00 (ref.)	1.00 (ref.)
Living alone	1.88 (1.69–2.09)^***^	1.42 (1.26–1.59)^***^	1.34 (1.20–1.51)^***^

Source: Data derived from the 1998–2014 NHIS–NDI record linkage study.

^a^ Age–year-adjusted model is the Cox proportional hazards model adjusted for age and survey year.

^b^ Sociodemographically adjusted model is adjusted for age, survey year, gender, race/ethnicity, nativity/immigrant status, education, poverty status, housing tenure, and region.

^c^ Fully adjusted model is adjusted for age, survey year, gender, race/ethnicity, nativity/immigrant status, education, poverty status, housing tenure, region, self-assessed health status, activity limitation, psychological distress, BMI, smoking status, and alcohol consumption.

^***^ *p*<0.001, ^**^p<0.01, ^*^p<0.05.

## Discussion

Our study contributes to the existing literature by using a nationally representative data set with 17 years of mortality follow-up and by adding to the evidence on the association between social isolation and risks of all-cause and heart disease mortality in the United States. The relative risk of all-cause and heart disease mortality was significantly higher among working-age adults living alone at the baseline than among those living with others even after controlling for a number of sociodemographic, health status, and behavioral characteristics. Regarding heart disease mortality, the relative risk of living alone was greater than that for all-cause mortality. The relative all-cause mortality risk of living alone was greater for working-age men at the baseline and non-Hispanic Whites, compared with that for working-age women and racial/ethnic minorities, respectively.

A number of biological and behavioral mechanisms underlying the relationship between social isolation and mortality have been reported.^3,39^ Social isolation or its markers such as living alone can increase the risk of mortality by provoking inflammatory processes, increases in C-reactive protein, elevated blood pressure, or by decreasing adherence to medical treatment.^39^

Living alone can also influence all-cause and cardiovascular mortality through other mechanisms such as socioeconomic disadvantage, health-risk behaviors (smoking, obesity, physical inactivity, inadequate sleep, and poor diet), poorer physical and mental health, and reduced access to care.^39^

In our study, controlling for sociodemographic, behavioral, and baseline mental and physical health factors decreased the relative risk of all-cause mortality among adults living alone by 20% from 1.45 to 1.16 and of heart disease mortality by 27% from 1.83 to 1.33. In our study cohort from 1998 to 2014, adults living alone had a 28% higher smoking rate (28.4% vs. 22.2%) and 9% higher alcohol consumption (71.4% vs. 65.6%) than those living with others.

Since mortality from various chronic and communicable diseases is a function of both incidence and patient survival, differential access to care plays an important role in determining patterns in all-cause and cause-specific mortality among individuals living alone and living with others. In our study cohort, compared with those living with others, adults living alone were 6% more likely to lack health insurance (20.1% vs. 19.0%), 5% less likely to have a usual source of care (79.3% vs. 83.5%), and nearly two times more likely to delay seeking care due to cost (20.0% vs. 9.8%).

Our study is consistent with previous studies on the association of living alone with all-cause and heart disease mortality.^3,9–14^ A meta-analysis of 70 studies found that the average effect size of living alone was 1.55 times higher in the age- and gender-adjusted model, and 1.32 times higher in the fully adjusted model, compared with living with others.^3^ The fully adjusted HR of 1.16 for all-cause mortality based on the 17-year follow-up in our study is slightly higher than that found in this meta-analysis study. This could be explained by the fact that our study uses a longer follow-up time (1998–2014), younger age group (18–64 years), and a larger and different set of confounders including sociodemographic, health status, and behavioral-risk factors.

Jensen et al.^9^ found that the relative risk of cardiovascular disease mortality for adults living alone was 1.36 times higher than that for adults living with others, which is similar to our finding for heart disease mortality (HR=1.33). For the gender-specific models, our study found that men living alone had greater relative mortality risk and lower survival probability than women living alone, consistent with findings in previous studies.^11,12,14^ Regarding race/ethnicity-specific models, although one recent study among adults aged 30 years or older, using a social network index, found that non-Hispanic Blacks had a higher mortality risk than non-Hispanic White,^20^ we found that non-Hispanic Whites living alone had a greater mortality risk than non-Hispanic Blacks.

This inconsistent findings might arise from the use of different measures of social isolation and age group. Despite variations in social isolation measures and covariates selected, our findings are compatible with previous studies using a composite measure of social isolation or loneliness that found a similar mortality risk ranging from 1.26 to 1.29^3,19^ and a greater mortality risk for men.^17,20,40^

As for the public health priority, the 2020 COVID-19 pandemic inevitably has encouraged self-isolation, social distancing, and teleworking as an effort to prevent spreading the virus. During the pandemic, vulnerable populations including individuals with low income, the elderly, and the racial/ethnic minorities would be at even higher risk for social isolation as well as COVID-19, given inequalities in access to resources, lack of teleworking availability, and the digital divide.^41–43^

Given lack of evidence regarding the most effective interventions, more theory-driven well-designed social interventions are needed.^43,44^ It highlights the need for greater resources for data collection, surveillance, and research on the association between social isolation, health, and mortality,^43^ considering NHIS has not included various measures of social isolation such as the social network index or loneliness except living alone. Public sectors including education, housing, and health need to set a policy agenda to improve social connectedness such as strengthening ties to community-based networks and resources or improving access to mental and behavioral health services through telehealth or remote communication technology.^39,41,43^

### Limitations

This study has limitations. First, our study only contains the NHIS sample eligible for linkage to the NDI. Excluding samples ineligible for linkage may lead to biased mortality estimates. To address this bias, we used the adjusted original sampling weight to account for the NHIS–NDI mismatches.^36^ Second, our findings may be affected by the omitted-variable bias. Although our Cox regression models were controlled for self-reported health status, BMI, activity limitation, psychological distress, and physical health status, there could be other potential confounders.

Third, all the covariates in the NHIS–NDI database were time fixed at the baseline as of the survey date. Several of the covariates such as SES, health status, behavioral-risk factors, and living alone could have varied over the long mortality follow-up period of 17 years, which would have influenced their estimated impacts on all-cause and heart disease mortality. Future studies need to evaluate the temporal robustness of living alone patterns in all-cause and cause-specific mortality using longitudinal data sets with time-varying covariates. Sixth, living alone might not fully capture social isolation and loneliness but it could be a good starting point for understanding an individual's social support.^45^

## Conclusions

In a nationally representative study with 17 years of mortality follow-up, we found that U.S. working-age adults living alone (i.e., experiencing social isolation) at the baseline had statistically significantly higher risks of all-cause and heart disease mortality than those living with others. Specifically, living alone was associated with 16% and 33% increased risks of all-cause and heart disease mortality, respectively, even after accounting for differences in a wide range of socioeconomic, demographic, behavioral, and health characteristics. The association between living alone and all-cause mortality was more pronounced among men and non-Hispanic White adults, compared with women and racial/ethnic minorities.

## Human Participant Protection

The study was exempt from institutional review board approval as it utilized a deidentified public use data set.

## Disclaimer

The views expressed in this article are the authors' and not necessarily those of the Health Resources and Services Administration or the U.S. Department of Health and Human Services.

## Abbreviations Used

AIANs: American Indians/Alaska Natives
APIs: Asian and Pacific Islanders
BMI: body mass index
CI: confidence interval
HR: hazard ratio
HRSA-OHE: Health Resources and Services Administration—Office of Health Equity
K6: Kessler 6
NCHS: National Center for Health Statistics
NDI: National Death Index
NHIS: National Health Interview Survey
ORISE: Oak Ridge Institute for Science and Education
SPD: serious psychological distress
